# Examining the variations in the implementation of interventions to address stillbirth from the national to subnational levels: experiences from Uganda

**DOI:** 10.1186/s12961-022-00928-w

**Published:** 2022-11-04

**Authors:** Eric Ssegujja, Michelle Andipatin

**Affiliations:** 1 Department of Health Policy Planning and Management, School of Public Health, College of Health Sciences, Makerere University, Kampala, Uganda; 2 School of Public Health, Faculty of Community and Health Sciences, University of the Western Cape, Cape Town, South Africa; 3 Department of Psychology, Faculty of Community and Health Sciences, University of the Western Cape, Cape Town, South Africa

**Keywords:** Stillbirth, Policy translation, Implementation context, Policy adaptation

## Abstract

**Background:**

The current global burden of stillbirth disproportionately affects regions such as sub-Saharan Africa, where Uganda is located. To respond to this burden, policies made at the national level were diffused from the centre and translated into service delivery at the district level, which is charged with implementation under the decentralization of health services arrangement. Variations emerge whenever policy recommendations are moved from national to subnational levels, with some aspects often lost along the way. Tools are available to facilitate knowledge of determinants of policy and innovation implementation within the healthcare system. However, the extent to which these have been applied to explain variations in implementation of interventions to address stillbirth reduction in Uganda remains scant. The aim of this article was to examine the variations in the implementation of interventions to address stillbirth from the national to the subnational levels in Uganda using the Consolidated Framework for Implementation Research (CFIR).

**Methods:**

The study adopted a qualitative case study design. Data were collected from a purposively selected sample of key informants drawn from both the national and subnational levels. All interviews were conducted in English and transcribed verbatim. ATLAS.ti was used to guide the coding process, which used a codebook developed following the CFIR domains as codes and constructs as sub-codes. Analysis followed a content analysis technique.

**Results:**

National-level factors that favoured implementation of interventions to address stillbirth included the desire to comply with global norms, incentives to improve performance for stillbirth reduction indicators for better comparison with global peers, and clear policy alternatives as process implementation advanced by champions. Variations at the subnational level revealed aspirations to address service delivery gaps which fell within maternal health routine standard of care and ongoing health systems strengthening initiatives. Coalescing existing networks around maternal and child health was a key mobilization factor for advocacy and programming, with a promise that the set targets would be operationalized at the subnational level. The key champions were defined by their official roles within the district health systems, which enhanced accountability. Feedback and reflection were distinguished from the national to subnational through joint assemblies and formal audit reviews, respectively.

**Conclusions:**

A heavy influence of the global events directed national-level adaptation of interventions to address stillbirth. Implementation context at the subnational level led to local adaptation and translation of policy provisions from the national level to suit the context, which to a greater extent explains the variations in the final content of policy provisions delivered.

## Background

Of the close to 2.6 million stillbirths that occur every year, the majority of cases happen around the time of delivery [1, 2]. Low-income countries typically constrained by resource limitations are disproportionately affected. Within Uganda, stillbirth burden stands at 9/1000 live births, with many categorized as fresh stillbirths reflecting deficiencies in the quality of maternal healthcare, especially around the time of delivery [3]. To address the challenge, tremendous progress stemming from the Millennium Development Goals (MDG) era has registered success in reducing perinatal mortality and continues to be pursued under the Sustainable Development Goals (SDGs), specifically goal 3.2 [4]. Some of the key successes have been policy changes to improve health facility service delivery, health workforce education and training, community demand-side factors, and drugs and essential commodities that have witnessed improvements in quality of health services and hence an increase in the number of facility deliveries [5, 6]. Nonetheless, variations continue to be experienced as policies move from the centre to subnational levels. To understand the underlying dynamics, tools are available to facilitate the identification of underlying factors. The Consolidated Framework for Implementation Research (CFIR) is one among many that have been applied extensively but seldom used for the case of stillbirth reduction efforts. It is a meta-framework that borrows from other implementation frameworks to provide constructs that can be used to explain theory development and verification of what works and under what circumstances [7]. It contains five domains which include the intervention, inner and outer settings, and characteristics of individuals and processes. While each domain may appear independent of the other, they are interlinked during implementation to produce outcomes. Its ability to facilitate interpretation of observations at multiple levels makes it suitable to understand variations in implementation of stillbirth reduction strategies at national and subnational levels. Currently, innovative interventions to improve the quality of maternal healthcare especially around the time of delivery are being promoted in different areas across the globe and implemented in countries including Uganda. This is due to many of the maternal deaths and stillbirths being registered around the time of delivery, and yet known evidence-based interventions delivered around this time provide the greatest opportunity to save many lives [8].

The targeted national levels efforts to address stillbirth reduction have been prioritized in policy with the hope that transfer processes will see them translated into service delivery at the subnational level. Policy recommendations have highlighted several evidence-based interventions such as scale-up of basic and comprehensive emergency obstetric care services, periconceptional folic acid supplementation, prevention of malaria during pregnancy and improved detection and management of syphilis during pregnancy to address known stillbirth risk factors [9, 10]. Many of the proposed interventions have been or continue to be piloted in different parts of the world involving both low-cost and resource-intense interventions [11, 12]. As pilot results start to trickle in, so do the concerns over their effectiveness in reducing the stillbirth burden. A trial in the United Kingdom, which piloted interventions responding to known stillbirth risks through the creation of awareness among pregnant women regarding foetal movement, revealed that despite controlling risk factors for now, the intervention did not affect stillbirth occurrence [13]. Relatedly, the baby birth trial conducted in an Indian province of Utter Pradesh that aimed at piloting a WHO Safe Childbirth Checklist (SCC), another known evidence-based tool, revealed that despite the adoption of positive essential birth practices, it did not have an effect on stillbirth reduction in either the intervention or control group [14]. The experience of piloting the same in Uganda showed that although the SCC was piloted in both control and intervention sites, most reductions in stillbirth were registered among intervention sites where additional evidence-based tools were implemented, reflecting the low effect of the SCC in reducing stillbirth [15]. Application of the same tools in another Indian context of Rajasthan revealed that the SCC was an effective intervention with the potential to avert intrapartum deaths, with most reductions occurring in the prevention of stillbirths [11].

Whereas most of these experiences are from controlled settings, the results point to the importance of contextual factors in influencing implementation outcomes [16]. It is not yet known how such initiatives can play out when delivered through routine standard care services. Experiences of variations in the package from design to implementation, especially in maternal and child health (MCH) interventions, have long been established [17]. The implementation context has been advanced as one of the factors that can account for this. Detailed information on the exact contextual factors that make it difficult or influence implementation outcomes, however, remains scant. Drawing on experiences from elsewhere, contextual factors general to the health system and specific to context have been advanced as reasons for the observed differences in outcomes [18, 19]. These include the limited resources, which leads to a lack of inputs and infrastructural shortcomings that make it difficult to implement policies according to plan as they move from national to subnational levels. Earlier work from Uganda revealed that the narrow decision space was an impediment to implementation, [20] coupled with the managerial behaviours and practices of some of the health systems managers [18]. Even where implementation proceeds according to plan and behavioural change is successfully impacted, complexity issues may limit positive outcomes on intended indicators such as stillbirth [14].

Uganda adopted a mix of policy options to address maternal mortality and subsequently stillbirth reduction. These cover a wide array of health system building blocks to aid in realignment to address risk factors [17]. Among policies targeted were human resource for health improvements, prioritization of recruitment and deployment of rare cadres such as anaesthesiologists, having physicians as in-charges at the health centre IV (HCIV) level, designating a senior nurse as the assistant district health officer (ADHO) to oversee implementation of MCH services within the districts, and prioritization and recruitment of midwives as in-charges for HCII to oversee delivery of maternal health services at that level. Policy options targeting health systems efficiency included the addition of another layer (health subdistrict) below the district level under the decentralization arrangement specifically to oversee the delivery of maternal health services. In addition, HCIVs were to be operationalized to deliver comprehensive emergency obstetric and neonatal intensive care (CEmONIC), which necessitated equipment with a functional operating theatre complete with blood transfusion services, while HCIIIs were equipped to deliver basic emergency obstetric care (BEmOC) [21]. Policies targeted at service delivery improvement reflected boosting of antenatal care (ANC) attendance, promotion of facility deliveries under skilled attendants, delivery of services along the reproductive, maternal, newborn, child and adolescent health (RMNCAH) continuum of care, and an improved referral system [22]. A number of initiatives to address demand-side barriers such as male involvement in reproductive and maternal health, addressing distance to health facilities and discouraging delivery by traditional birth attendants (TBAs) were also prioritized. Other policy options were in the areas of improving availability of reproductive and maternal health medical commodities and improvements in data capture to improve visibility of stillbirth burden. This was reflected in the inclusion of stillbirth as a notifiable condition within the surveillance systems and stillbirth as a quality-of-care and performance indicator for health facilities and districts, respectively, among others. However, translation of all these interventions at the final points of service delivery varied depending on prevailing contexts.

The above experiences reflect variations in implementation as policies move from the national to subnational levels. This calls for a critical review of the process factors likely to account for this observation. Although the greater influence of contextual factors than the intervention package in determining the observed outcomes is acknowledged, a consensus is yet to be reached regarding the exact contextual factors that may exert the most influence over outcomes [23]. This falls short of the premise underpinned by the diffusion of innovation theory. Policy transfer theories follow these underpinnings where it postulates that once an idea or policy aspect is introduced into a new setting, actors through their actions and relational networks will facilitate its adoption across the different levels of implementation. Hence, to answer these questions, this study was conducted among national-level policy-makers, policy implementers and health partners. At the subnational level, the target study population comprised district health managers, health facility managers and frontline health workers. Using the CFIR, the aim of the study was to examine the factors accounting for variations in implementing interventions to address stillbirth from the national to subnational levels in Uganda.

## Methods

### Study design

Details about the study design have been explained elsewhere [3, 24]. Briefly, the study adopted a qualitative case study design. A qualitative case study approach was preferred because of its exploratory nature that provides an in-depth understanding of the “how” and “why” which facilitate a deeper understanding of the phenomena and context. It was conducted at both the national and subnational levels, with one district acting as a case study for subnational-level implementation. The CFIR was utilized at both the national and subnational levels to assess the implementation of interventions to address stillbirth. It was applied as a deductive codebook for content analysis and as a guide to facilitate the interpretation of results.

### Study setting and population

The study was conducted in Uganda. At the time of the study, components of the recommended interventions to address stillbirth were at varying levels of implementation and adaptation. For example, operationalization of birth and death registration, which was intended at the recording of all stillbirth happening at the community level, had not been fully realized by the National Identification & Registration authority (NIRA). Similarly, the community health extension worker (CHEW) strategy meant to operationalize the minimum recommended eight [8] ANC contacts during pregnancy was not fully operational. On the other hand, the country was in full operational mode for other policy components such as stillbirth notification, operationalization of CEmONIC and BEmOC, and conducting the maternal and perinatal death surveillance and response (MPDSR) at the health facility level, albeit with some challenges. The country opted for a stepwise approach towards implementation of these recommended interventions rather than adopting all at once. At the subnational level, the study was conducted in Mukono district located in central Uganda and bordering Kampala, the main commercial and capital city of the country. At the time of study conceptualization, it was one of the districts with the highest burden of stillbirth as reflected in the Annual Health Sector Performance Report (AHSPR) 2015/2016. The study population at the national level included key informants drawn from the MCH policy communities. At the subnational level, the study population comprised frontline health workers and health managers at different levels of health system functioning including the facility level, subdistrict level and district health office, where members of the district health management team (DHMT) were targeted.

### Sample and sampling procedure

The final study sample comprised 36 respondents, with 20 drawn from the national level and 16 from the subnational level. Within the results section, national-level interviews are labelled with the initials “NLI”, while interviews conducted at the subnational level are labelled with the initials “SNLI”. Respondents were purposively identified through a consultative process with the help of informants familiar with MCH policy implementation processes. This was augmented with a snowball criterion which was also applied to arrive at additional respondents after commencement of the study, where respondents were asked at the end of each interview to identify any and recommend potential respondents that were familiar with the study subject. A stepwise process was employed to enhance inclusion and arrive at the final potential respondents. It involved drawing a list of all entities from which potential respondents, particularly those familiar with MCH policy implementation, were obtained. Telephone numbers of potential respondents were then obtained, whereupon which contact was initiated, study objectives explained and a request to participate extended with a potential day of interview requested. A total of 23 national-level respondents were approached for participation, out of which 20 were interviewed, while at the subnational level, 16 respondents were interviewed. We did not pursue further interviews after these, since no new data were emerging. On the day of interview at the health facility, further explanation was given and informed consent obtained. Further consultations were then performed to identify more potential respondents in cases where we found that the pre-identified potential respondent was not available during the duration of the study.

### Characteristics of the study sample

Overall, 17/20 national-level respondents were female, drawn from the Ministry of Health (MoH; 5), nongovernmental organizations (NGOs; 4), professional associations (6), private not-for-profit health facilities (2), academia (2) and private for-profit entities (1). Their places of work were not mutually exclusive, as some doubled as working in academia, affiliated to professional associations and linked to either private not-for-profit or private for-profit entities. Subnational-level respondents included members of the DHMTs (3), hospital (6), HCIV (5) and HCIII (2). These varied by membership to include medical officers, nurses and midwives.

### Data collection

Prior arrangements were made where potential respondents, particularly those at the national level, were first approached by telephone, where the aims and objectives of the study were explained. The process involved conducting key informant interviews using semi-structured interview guides to collect data at both the national and subnational levels. Different tools were used for the national- and subnational-level interviews. Their development was guided theoretically by policy translation and diffusion by literature [25, 26]. The national-level tool explored national-level political prioritization of global health campaigns, while the subnational level tool explored policy translation experiences reflected in health workers’ experiences, the role of facility management and individual health workers in translation of policy goals, workplace context and service users’ characteristics. Interviews were conducted face to face from March to June 2019 at the respondents’ places of work following agreement on time and day when interviews were to be conducted. The interviews were conducted by the first author (ES), assisted by two female graduate-level research assistants. Interviews were recorded using a digital audio recorder, with field notes taken during the process. Overall interviews lasted between 45 minutes and 1 hour, and the interview guides used have been explained elsewhere [3, 24]. At the national level, two call-backs would be made before a potential respondent would be replaced, while at the subnational level, none of the contacted potential respondents declined to participate.

### Conceptual framework

The study utilized the CFIR, one of the most commonly used implementation science frameworks [7]. The framework was applied deductively to analyse and understand the underlying factors for variations in implementation at both the national and subnational levels of implementation. It consists of five domains that interact iteratively in complex settings to influence implementation effectiveness. The five domains include (1) the intervention characteristics, (2) outer setting, (3) inner settings, (4) characteristics of individuals involved and (5) process implementation. Within each domain, several constructs explain different assumptions, expectations, beliefs, factors and theories behind the effective implementation of the intervention. It postulates that the implementation of interventions is influenced by the different domains working through multiple constructs to determine outcomes. Conceptual frameworks are ideal for analysing complex interventions, for they explain variables either graphically or in narrative form that influence a phenomenon of interest, thereby increasing the generalizability and interpretability of results [27–29]. The CFIR was preferred for its flexibility to be tailored to the context for this study and its ability to explain the nonlinear complexities surrounding the translation of interventions from the national to subnational levels of the health systems. Details of the domains, their definitions and specific constructs are further explained in Table 1.

**Table 1:** CFIR [7]

	Domain	Definition	Related constructs
1	Intervention characteristics	Features of the intervention that might influence its implementation	Eight constructs comprise this domain: (1) intervention source, which refers to perceptions of whether intervention is internally or externally developed; (2) evidence strength—perception of quality and validity of evidence; (3) relative advantage—perception of advantage of implementing intervention vs available alternatives; (4) adaptability—degree to which intervention can be tailored or refined to meet local needs; (5) trialability—ability to test intervention; (6) complexity—perceived difficulty of implementation; (7) design quality—perceived excellence in how intervention is packaged; and (8) cost—expenses associated with implementation
2	Outer setting	Features of external context and environment that might influence implementation	Four constructs form this domain: (1) patient needs—extent to which patient requirements and barriers are known and addressed; (2) cosmopolitanism—extent to which organization is networked with other organizations; (3) peer pressure—competitive pressure to implement from peers; (4) external policies and incentives—external strategies to spread intervention
3	Inner settings	Features of the implementing organizations that might influence implementation	12 constructs relate to this domain: (1) structural characteristics—social architecture and size of organization; (2) networks and communication—webs of interlinkages within an organization; (3) culture—norms, values within an organization; (4) implementation climate—absorptive capacity for change; (5) tension to change—extent to which stakeholders perceive current situation as intolerable; (6) compatibility—tangible fit between meaning and value of intervention; (7) relative priority—shared perception of importance of implementing intervention; (8) organizational incentive—extrinsic value of implementing intervention; (9) goals and feedback—communication of goals to staff with feedback aligned to the goals; (10) learning climate—individuals feel safe to try new methods with sufficient time for reflective thinking and evaluation; (11) readiness to implement—tangible and immediate indicators for organizational commitment to implement intervention; and (12) leadership engagement—commitment, involvement and accountability of leaders to implementation
4	Characteristics of individuals involved	Characteristics of individuals involved in implementation that might influence implementation	Five constructs form this domain: (1) knowledge and beliefs about intervention—attitudes and values placed on the intervention; (2) self efficacy—individual beliefs in their own capacity to execute the intervention; (3) individual stage of change—phase of progress towards skilled, enthusiastic use of intervention; (4) individual identification with organization—individual’s degree of commitment and relationship to the organization; and (5) other personal attributes—broad to include other personal traits such as intellectual ability, tolerance of ambiguity, etc.
5	Process implementation	Strategies and tactics employed that might influence implementation	Eight constructs form this domain: (1) planning and execution—developing tasks for implementing intervention; (2) engaging—involving appropriate individuals; (3) opinion leaders—individuals within organization with formal and informal influence over attitudes of others; (4) formally appointed internal implementation leaders—individuals with formal responsibility for implementing intervention; (5) champions—individuals who dedicate themselves to support and market intervention; (6) external change agents—individuals affiliated with external entity who formally influence intervention; (7) execution—accomplishing the implementation according to plan; (8) reflection and evaluation—quantitative and qualitative feedback about the implementation process

### Data analysis

To facilitate the analysis of variations in implementation between the national and subnational levels, transcripts from the national-level sub-study [3] and subnational-level sub-study [24] were integrated. The national-level context involved translation of policy recommendations from the global campaigns into national-level policy provisions, while subnational context involved translation of national policies into maternal healthcare service provision. This was followed by a codebook development exercise which followed domains and constructs from the CFIR. Specifically, each of the domains was adopted as a code, with constructs also adopted as sub-codes. Thereafter, transcripts were exported into ATLAS.ti, a qualitative data management software program [30]. A framework analysis technique was adopted as an analytical approach. It was preferred for its ease of configuration with a deductive coding technique [31, 32]. After familiarization with the data, coding was deductively conducted following these CFIR domains and specific constructs under each domain. A coder would identify textual data relating to specific constructs, highlight them and then attach the data to a specific sub-code corresponding to a particular construct. The team then developed an analytical framework, having familiarized themselves with the data during the coding process [33]. At the end of this process, query reports were produced for each of the constructs, followed by a manual pile-sorting exercise leading to the grouping of text with similar meaning under unique piles in a data-charting exercise guided by the applied analytical framework. The team then read through each of the textual data once more to identify underlying meaning in a data interpretation exercise, which led to identification of factors related to variations in implementation of specific policy recommendations between national and subnational levels. Typical quotes are used when presenting the final results under each domain and construct. We present results based on the underlying meaning as it related to the identified constructs within the applied CFIR. Finally, we follow the Consolidated Criteria for Reporting Qualitative Research (COREQ) when reporting the qualitative results [34]. Detailed information about the study team has already been reported elsewhere [3, 24].

## Results

### Intervention characteristics domain

#### Intervention source

From the national perspective, a desire to comply with global MCH targets appears to explain the national efforts to address the stillbirth burden. This motivation was not exclusive to stillbirth reduction, but can also be traced across several other reproductive maternal, newborn and child health (RMNCH) indicators. Within this context, a focus on stillbirth reduction was an extension of national strategies to address the low-performing RMNCH indicators. Acknowledgment of external support to tailor national objectives was a common theme across respondents:

*When we adopt or get guidance from, let’s say, the World Health Organization on how to manage newborns or maternal health issues, we, through the Ministry, partners, professional medical association bodies can sit down and comb through and see how to contextualize these guidelines.* (NLI019)

At the subnational level, the likely causes and burden of stillbirth were familiar to the service managers and providers. These were addressed through already existing interventions delivered through the routine standard of care. There was a general feeling that efforts to support addressing stillbirth reduction were welcome and long overdue. Tailoring national efforts to fit local context is what was adopted on a case-by-case basis at the final point of service delivery. The widely held perceptions were that triggers for most stillbirth risks originated from the community and were due to delayed or late referrals. This knowledge of the causes was a common observation among respondents.

#### Evidence strength and quality

The global connection of the intervention source had an influence on the nature of evidence presented. The observation that an exclusive stillbirth indicator was omitted in national-level statistics seemed to have created discomfort among advocates. A feeling that the high perinatal deaths were being disproportionately skewed by stillbirth was another point of concern. The adoption of evidence from the global campaign found a soft ground to galvanize national-level support to join the efforts in addressing the problem at the national level. Nonetheless, thoughts on insufficient data persisted, prompting support for existing deliberate efforts to improve data systems.

*We are making sure that that is part of the strengthening we are talking about. So we are making sure that once these partners are also working with the districts—and we are reporting through District Health Information Software 2 (DHIS2), and so once we do that, we are strengthening the DHIS2. The annual reports that people talk about have much better data than what has been previously happening, so we are seeing that as a chance in the activities that we have.* (NLI008)

Variations at the subnational level with regard to evidence strength were reflected in the way that most health workers involved in the delivery of MCH services were familiar with and had recent experiences of encounter and management of mothers with stillbirths. The belief that addressing stillbirth was long overdue was widely shared. National-level prioritization found familiar context, contributing to its acceptance at the subnational level.

*My experience with stillbirth is that it has been on the rise, especially the fresh stillbirth, because for the macerated we have been able to teach the mothers what causes it and now at least they have improved. Then for the fresh stillbirth, it has been rising in our hospital, because in Mukono this is the only big hospital around and we get many referrals in.* (SNLI025)

#### Relative advantage

Closely related to the relative advantage was evidence used which reflected an interconnectedness between the maternal mortality risk factors and stillbirth risk factors. From the national perspective, prioritizing stillbirth meant boosting MCH systems and service delivery improvement efforts since they shared similar risks and possible interventions to address risks. Besides, these global campaigns were attracting funding, something the government embraced to reinforce its strategic objectives of systems strengthening. This would in turn respond to the persistent challenges of demand-side bottlenecks which had inhibited optimal utilization of available maternal health services to encourage facility deliveries under skilled attendants.

*We are talking of strengthening the system like in most districts where we have substantive district health officers who are the health managers in the district, we have the ADHO who manages maternal and child health and is also responsible for immunization, so through that angle most of the systems are okay, and then you look at the facilities where they are delivering. Now, the Ministry of Health, through a project called Expand on Maternity Wards in Health Centre IIIs and strengthen them and also skill through mentorship and courses.* (NLI007)

Maternal health was already a prioritized intervention at the subnational level, with utmost caution exercised to avert any maternal death. Since many of the strategies to address stillbirth were similar to those for maternal mortality, it presented a relative advantage for embracing stillbirth reduction within the ongoing MCH standard of care.

#### Adaptability

Maternal mortality was already a notifiable condition captured within the surveillance systems. This made the inclusion of stillbirth in the same surveillance system less complex. Besides, maternal death reviews were already an area of focus in national policies; therefore, adapting perinatal death reviews seemed to have made its prioritization much easier. At the subnational level, the district already had a designated officer in charge of MCH, and stillbirth reduction was added to the indicators the officer had to prioritize while at the facility level. The health workers followed national guidelines to report any stillbirth within 24 hours. Besides, it did not require setting separate guidelines for perinatal deaths reviews, but rather stillbirth inclusion among the cases to be reviewed:

*You see, the Ministry says that if you get a maternal death, they must be notified within 24 hours. Normally we notify them and we also notify the office of the DHO, but the most important thing is for us to find out the gap and address it.* (SNLI029)

#### Trialability

Stillbirth reduction efforts benefited immensely from evidence generated from maternal health projects implementing neonatal components. It is evident from these interventions that were later used to support advocacy, inform policy revisions and support the national-level rollout. Other evidence-based tools like the WHO SCC piloted by the East Africa Preterm Birth Initiative had results ready to inform policy modifications. More recently, during the adoption of WHO quality-of-care guidelines, the country selected 11 districts for trial purposes, as explained in one of the interviews:

*Currently, under the WHO, we have quality of care improvement for maternal and newborn, and we have adopted the WHO guidelines where we are emphasizing quality in maternal and newborn care as one of the strategies of reducing both maternal and newborn deaths. So we rolled out the guidelines, we have identified 11 planning districts that are going to represent those standards.* (NLI007)

Evidence of trial flexibility was observed in the study district. Previous MCH quality improvement interventions such as the Helping Babies Breathe (HBB) project were piloted in the area. Implementation experiences from the project became health workers’ capacity-building turning points. In addition, evidence from other within-country project pilots such as Saving Mothers, Giving Life (SMGL) piloted in other districts reinforced and informed district-level strategies to address stillbirth risk when lessons were disseminated outside the pilot districts. These were adapted into routine MCH services offered at the respective levels.

#### Complexity

Adopting strategies to address stillbirth at the national level was viewed as a complex process. Initiatives targeted at improving the quality of RMNCH services were intertwined with chronic health systems challenges impeding quality service delivery. Therefore, planning to address stillbirth had resource implications for which attention to outstanding challenges was needed. To streamline prioritization, MoH opted to first respond to facility-based fresh stillbirth as they confronted other chronic health systems challenges:

*To tackle these issues, you also need to address the deep-seated health system bottlenecks. For instance, we know that for one to handle these mothers and babies well, you need to have a skilled midwife or doctor who is available on site. So, over the years, the Ministry has been doing its best to see how they can increase the staffing especially of midwives at the public facilities.* (NLI019)

Contextual complexities inherent at the subnational level often played out in efforts to address stillbirth. Supply-side bottlenecks such as inadequate staffing affected demand-side factors like its negative effect on care-seeking. The demand-side barriers to service access for which health workers had little influence continued to persist, such as delays in healthcare-seeking and dysfunctional referral systems among others.

*You see, the Ministry even abolished traditional birth attendants, but they are still operating and they are taking a lot of mothers; when compared to the number of mothers coming to our health facilities, the numbers are still big. And among the reasons is the issue of understaffing … we need to address the knowledge gaps in the communities and also among the health workers.* (SNLI021)

#### Design quality and packaging

The rollout of some interventions was preceded by streamlining misaligned aspects within the policy. Many of the fresh stillbirths happening from within the health facilities were due to late referrals. Addressing policy gaps became important to respond to these cases, as explained in one of the interviews below:

*The second thing that is happening now is that we are revising some areas in policy that are going to help streamline referrals. So, the referrals hospital’s policy is going to be critical. The third one is the ambulance system. The new ambulance policy is also going to help in the new referral policy, so in a way, certain things are more in line to improve at least in the policy arena.* (NLI008)

Subnational-level challenges reported included those arising from design quality and packaging incompatibilities. A case in point was the conduct of perinatal death reviews amidst scarcity of tools/forms, with low appreciation as compared to maternal death reviews. Subnational response targeted this among challenges as reflected in an interview with a respondent:

*Even when we were going to do this result-based financing evaluation, among the aspects they are looking at was that maternal and perinatal death reviews (MPDR), they were looking at whether the facility has the form and whether the committee sits. So, we are just even struggling to download the forms and put them in the files. That one is not well appreciated.* (SNLI027)

#### Cost

Country-level efforts were boosted with the availability of funds to support different pilots with a neonatal health component. Later, this was an important resource in informing policies responsive to stillbirth reduction.

*To realize this vision, the Ministry has also adopted certain programmes like the results-based financing which is focusing on maternal-child health issues. So that is a focused effort by the Ministry which understands that this is a problem and we need to have some kind of focused effort to address it.* (NLI016)

After analysis, no variations in implementation were observed between national and subnational levels, where similar resource opportunities enabled rollout. The World Bank support to national-level MCH improvement goals through the Uganda Reproductive, Maternal and Child Health Improvement Project (URMCHIP) was a typical case. It operationalized results-based financing (RBF) that compensated outputs for preselected indicators, of which some related directly to the country’s efforts to address stillbirth. These include the conduct of MPDSR, among others.

### Outer settings

#### Patient needs and resources

Demand-side barriers impeding facility deliveries had been at the core of MoH strategies to address maternal mortality. Stillbirth campaigns were initiated to address the distance to health facilities and improve the quality of maternal healthcare services through the operationalization of basic and comprehensive emergency obstetric care and improved referral systems.

*A lot of effort has been done to encourage mothers to come and deliver in health facilities, but when they come they are losing their children. Antenatal coverage we are saying has gone up, but when you look at the fourth antenatal care, coverage is far below compared to the first ANC and is still below standard, so all those are factors that are leading to the death of our newborns.* (NLI007)

There was a need to bring services closer to the users, including comprehensive emergency obstetric care with linked services to improve the referral system at the subnational level. It emerged that referrals of high-risk mothers for delivery in tertiary facilities with the ability for blood transfusion were often not welcomed by such mothers preferring to deliver in nearby facilities.

*Looking at the stillbirths alone, most of the cases we get here are fresh stillbirths and those are mothers that are usually been referred from the community … and when we assess, they were being referred from these clinics, and one that came with a ruptured uterus at 28 weeks, and we could not save the baby.* (SNLI021)

#### Cosmopolitanism

At the national level, Uganda was actively involved and part of the global efforts to address maternal mortality. These events predate the onset of the MDG era during the early 2000s which triggered campaigns to reduce maternal and neonatal mortality by 2015. Cumulative efforts translated into national acceleration of implementation to achieve the MDG set targets. The post-MDG era witnessed the extension of similar efforts, with Uganda being a signatory to several global commitments. Maternal health, and by extension stillbirth reduction prioritization, was a benefit which had to be reflected in ways the interventions were implemented. At the subnational level, aggregated stillbirth numbers were part of indicators for monitoring health facility-level and district-level performance within the annual health sector performance reports. A high stillbirth burden would reflect poor performance which would be compared in relation to other health facilities and districts. Health facilities were networked through a localized referral system, which in outlook appeared interest-driven. Strategies to reduce stillbirth were communicated through these existing relations.

#### Peer pressure

The desire to keep up the pace with the set Every Newborn Action Plan (ENAP) targets by 2020, 2030 and 2035, together with other countries where Uganda was one of the ENAP countdown countries, necessitated close monitoring of set indicators to signal progress towards these targets. At the regional level, related obligations arose from the regional treaties that exerted pressure on the country to act in improving maternal health services for which stillbirth reduction was embedded:

*There are certain things that the East African Region has asked for which I think are good, but for the implementation, it’s up to the Ministry to decide. They are implementing some of these regional approaches; for example, there is a scorecard and we all contribute to that scorecard. Then there are regional issues around how training doctors should be done.* (NLI008)

At the district level, high numbers of stillbirth signalled poor performance compared to peers. At the facility level, the same high numbers of stillbirth signalled poor quality of MCH service when compared to other health facilities within the district. This was complicated by MoH’s request that facilities register all stillbirth cases even when they originated from the community but reported to the health facility when affected mothers presented for management of delivery complications or postpartum care. This would be irrespective of whether they occurred within the health facility so long as they were within the catchment area and managed to reach the health facility. Some health workers from affected facilities expressed dissatisfaction with this decision.

*Yes, you can have the high numbers, but the Ministry says when the mother comes to you, start from there, but by the time they come we have nothing to do and you have to admit that this is an FSB [fresh stillbirth]….what I can say, if it is a fresh stillbirth, they should not put it in our book. At least let them get away of reporting such, say “referral in-FSB” so that they don’t pin the hospital.* (SNLI025)

#### External policy incentives

Global appraisal of evidence regarding interventions with the highest impact coupled with mapping of policy areas that required realignment to address the problem appear to have offered direction to national-level processes. Redirecting resources from global initiatives in support of intervention implementation for programmes with neonatal components all contributed to incentives directed at favouring national stillbirth reduction action plans. Evidence to policy was handled at the Ministry level during steering committee meetings, where different players including representatives of bilateral and multilateral agencies were given an opportunity to present and deliberate on the evidence available before informing and adoption into policy. Despite these contextual factors at the time, reservations were expressed with regard to the influential role of partners directing the national response:

*The major way they have done it is through the steering committee meetings that they have at the Ministry level where they bring all key players, people who are doing the work. If I would just mention a bit of a challenge in my opinion that I think should be improved is that sometimes it is partner-driven, where a partner will front support [to] have these meetings, and so if the partner runs out of funds or he is no longer existing, then the meetings kind of stagger.* (NLI020)

At the subnational level, the performance rewards through the URMCHIP led to rapid scale-up of some interventions. Efforts to improve stillbirth data quality to create visibility to the hidden burden partly informed the MoH establishment of requirements for all health facilities and districts to have all stillbirths captured through the surveillance system and notified within 24 hours. This in itself added another accountability layer to improve reporting and adherence to set guidelines such as perinatal death reviews and documentation.

### Inner settings

#### Structural characteristics

Maternal health services within which stillbirth reduction strategies were embedded were a mobilizing factor among multiple stakeholders including donor agencies, researchers and professional associations. Their working relationships and networks were further strengthened in the MDGs and in pursuit of the national ENAP targets. MoH then guided the policy direction and overall stewardship while moving these targets from the national to subnational level. Similar networks exist at the subnational level through which service delivery is managed:

*The district health officer who is in charge of the health issues that happen in their respective districts, and it is through that channel that districts can get clear communication and feedback on those issues that are implemented at the Ministry level and also at the district level, because through the DHO and his team where the Ministry has a structure, among the district team members there is a maternal-child health focal person, and we know that through our experience that having a focal person to take lead on a particular programme issue helps to drive the process in a catalytic manner.* (NLI019)

#### Networks and communication

Established networks at the national level coalescing around the MCH cluster and other subcommittees such as the newborn steering committee with shared ENAP goals operationalized through the RMNCH investment case existed. These networks included multi-representation from stakeholder networks like professional associations with direct access to policy-makers. The strong working relationships were embraced within the MoH systems strengthening efforts to address chronic challenges identified and advocated for by partners for a long time.

*We know that the Ministry of Health is the overseer, is the team lead, is the driving force that ensures we comply with the existing policies and guidelines and how they manage to see that this is being done through support supervision and these are done directly by the headquarters staff, people who sit at the Reproductive Health Division here in Kampala, but since they are few, they also work through channels like regional-level structures.* (NLI018)

At the subnational level, tertiary facilities had already established working relationships through which they reached out to lower-level public health facilities, private facilities and TBAs within their catchment areas. At this level, the MCH service provision ecosystem was contextually different, for which they had to adjust service provision. Contextual challenges included private providers withholding mothers far too long before referrals, TBAs remaining active and major players in the referral system with some delays attributed to their role. Facilities initiated call-backs for lower-level service providers to follow up on referred cases to establish how they were responding to identified gaps. At the top were the district management teams that ensured all aspects were well coordinated to streamline communication and avoid duplication.

*One thing that the districts over time have been strengthening is the clear stakeholder engagement in their respective districts to ensure that they at least don’t have a lot of duplication or things happening under their nose that they are not aware of, because they are the gatekeepers and are responsible to give feedback at Ministry level as well.* (NLI015)

#### Culture

This construct was not explored in depth for both the national and subnational levels.

#### Implementation climate

MCH still held very strong norms originating from the MDG era which were again reinforced within the post-MDG development agenda such as the SDG and universal health coverage (UHC) that looks at comprehensive coverage. The desire to improve specific MCH indicators including stillbirth reduction was already a need that was felt. Stillbirth reduction benefited from this, and since similar interventions had the potential for triple return on investment, its acceptance met little resistance. Support from global bodies and MCH stakeholders was another aspect that made the implementation climate favourable, with country offices engaged in direct national-level rollout:

*Globally we maybe adopt standards, having done a lot of research and benchmarking; then as a country we customize. We have a country office here, and so it is their role to come to the Ministry of Health, and they adopt those standards and roll them over to the implementing sites, and those are the districts and all facilities, and like for this case, the quality of care we have to scale up into the entire country other than having learning districts.* (NLI007)

Stillbirth remained a sensitive issue even at the subnational level, where MoH engaged in spot checks when numbers exceeded the regular threshold from what was routinely reported. Sharing similar strategies with maternal mortality reduction efforts made addressing stillbirths compatible in the context of a limited-resource setting and the existing policy environment.

*We got feedback when we sent our health management information systems report, and we indicated that we had 3–4 fresh stillbirths that month. We had a team from the Ministry that came to find out what the problem was. And that was about 8 months ago. But from the perinatal death audits we send, we don’t get feedback.* (SNLI021)

### Process implementation

#### Planning

Clear policy alternatives were evident at the national level, with strategic objectives highlighted in national policies, particularly the RMNCAH investment case as the overall guiding policy. The Reproductive Health Division within the MoH oversees and coordinates planning at the Ministry level. The national strategy to deliver stillbirth reduction was through the routine standard of MCH services. The consensus of delivery as reflected in policy guidelines took on an integrated approach where services were delivered along the continuum of care, with service quality enhanced through health systems strengthening.

*We plan together with the districts. We have our new plans, and then the Department of Planning has regional plan meetings to look at issues of budgeting for health and budgeting for all resources. We coordinate the partners. We also earmark resources to the district, but more so for a partnership. We look into the policy, and the implementer is the government. We set the guidelines and the protocols through maybe institutions like Mulago [national referral hospital], and the academia may help us come out with standards and protocols, which we distribute.* (NLI007)

Feelings emerged that current plans were not commensurate with population growth rates, with resulting pressure on existing maternal health services. Proponents of this view argued that current maternal health challenges including the stillbirth burden stemmed from the poor planning that did not match the current demand for services, as expressed in one of the interviews:

*The problem is the systems and not responding to our population growth. And because of that, our current system, our population is outstripping the services that we currently have, and so until we make sure that our population growth and our health system can balance out, we shall still have a lot of problems. So, I know there are approximately 1.7 million pregnancies every year, but our facilities are not designed to provide services for the 1.7 million. So, we need more functional facilities to be able to provide the high-quality services that they should be providing. We also know that our medical degree graduate numbers every year—if we continue with our training at the moment, we are at 3% annual population growth and we are nowhere near being able to reach the numbers that WHO recommended, so until we see that, it is going to be very hard.* (NLI008)

Suggestions were advanced regarding the need for holistic implementation, since most bottlenecks were already known and only impeded by the disjointed implementation of interventions.

*I feel we have the answers to what the problem is and where the problem is. The thing is reinforcing efforts to do these interventions holistically; [by] holistic I mean we know the three delays, and I really feel that covers and encompasses everything, where if the mother does not know when and how quickly to seek that care, and if at all she goes to the facility and does not get the quality of that care, then that is the beginning of all these problems.* (NLI019)

Subnational-level efforts were characterized by joint planning for district-level stillbirth response which was embedded in strategies to address maternal mortality. The DHMT planned for execution of strategies through the respective subdistricts, while at the facility level, the in-charge and maternity unit in-charge together with staff made plans during staff meetings.

*But we always talk during meetings, and they will talk about the quick referral here. When we go for the district meetings, we encourage them to refer quickly and monitor mothers well, but still, I don’t think I have any other thing because—apart from saying that we re-refer those very complicated cases which will also be a problem to the mother.* (SNLI026)

#### Engaging champions

Enthusiastic national-level champions were more defined by their passion for maternal health. Interest in stillbirth reduction appeared more as a spinoff from earlier efforts in maternal health advocacy and intervention implementation. It was more the maternal health champions that took on stillbirth advocacy. These oscillated between advocating for stillbirth reduction and falling back to their dockets of MCH service improvement. The concept of patient advocates in MCH is still very weak in this context outside HIV services. Influential subnational champions were more defined by their official positions held in career and professional ranks as understood through their job expectations. The ADHO is responsible for championing MCH causes and subdistrict in-charges, while facility in-charges, maternity unit in-charges and midwives were the champions at the facility level. These are supported by health workers attached to the maternal health unit.

*Our health workers do everything, if I may say, and ours (management) is to support their decisions and being able to implement them and support them in logistics, consumables and supplies. So they do a lot, especially in ensuring that the treatment is given, those preterms are reported to us, and doctors are called in to review. And also ensuring that when death has occurred, they convene a meeting and look through the gaps and address them.* (SNLI024)

#### Execution

Strategies to address the national-level stillbirth burden were adopted into policy for translation by subnational administration. Policy alternatives including the addition of stillbirth to the list of notifiable conditions, a focus on fresh stillbirths, incorporating perinatal death reviews within existing maternal death reviews, and adopting stillbirth as a district performance indicator reflected in the annual district league tables, among others, were considered. These were very clear national-level strategies with targets expected at specific time points. Extension of coverage for emergency obstetric care services was another clear policy direction for the overall maternal health quality with stillbirth reduction benefits as revealed during the interview:

*Needless to say, we need to look at strengthening emergency obstetric care, because once you are doing emergency obstetric care, then you are going to reduce the fresh stillbirths. So those are the key areas that we are kind of trying to address.* (NLI008)

Interventions to address stillbirth at the subnational level were embedded within routine MCH services. Execution of identified strategies in the study area benefited more from the daily experiences and feedback from meetings as well as guidance from the MoH policies. Health workers implementing interventions to address stillbirth did so within their routine standard of MCH care.

*You know, the MS would call for internal meetings. What we do is that we divide up and the doctor would say that today give as a CME [continuous medical education] topic. Still, there are daily reporting where we have a person in this department who is the focal person for children. I [also] keep checking on them, and if there is a loophole somewhere, we always give a general CME.* (SNLI026)

#### Reflection and evaluation

Platforms for reflection and evaluation existed at the national level, where participation varied. The annual joint review mission, an accountability platform for the health sector as a performance review mechanism, is conducted every year. Similarly, the annual national assembly on RMNCAH sets thematic targets for government and partners to pursue and supports their implementation, such as strengthening accountability mechanisms. Other platforms included the newborn steering committee with a mandate from MoH to advise on newborn survival, the MCH cluster, and health policy advisory committee (HPAC) meetings, among others. Specifically, the annual national assembly on RMNCAH offers a distinct platform where implementation feedback is shared with national- and subnational-level key stakeholders. Monitoring of district performance indicators is a platform for reflection and evaluation of the effects of policy on the burden.

*And we have an annual assembly where we sit and evaluate that [during] the first period, how have we performed and how can we make commitments to the coming year. This includes the ministries of gender and others because there are many contributors other than the health sector to health.* (NLI007)

*I know we do monitor because we have, like at the Ministry level, the annual performance report that brings out districts that have issues with either maternal deaths or stillbirths. So, there is a system that we are monitoring.* (NLI019)

Subnational-level platforms established from the centre such as the MPDSR, together with local initiatives including continuous medical education (CME) and staff meetings, existed to support reflection and evaluation. These are forums for discussing facility performance with regard to stillbirth burden in addition to district-level initiatives. They have evolved to include not only documented stillbirths but also near-miss cases. Results from formal reviews offered space for reflection on the service quality to identify gaps and tailor response.

*We have daily reminders and guidelines because we have senior people here, so they keep reviewing and reminding and then getting results to identify individual weaknesses and try to correct them.* (SNLI030)

*We take monthly, and whenever we finish the reports we give the feedback to our staff. Every Wednesday we have a meeting, so if we finish the report on Monday, on Wednesday we have to give them a report. Someone can ask what happened; then you tell them that we got a referral with a ruptured uterus.* (SNLI025)

## Discussion

This paper examined the variations in the implementation of interventions to address stillbirth at both the national and subnational levels in Uganda during the period between 2010 and 2018 when fieldwork for this study was conducted. Indeed, our results point to variations in implementation of interventions at both levels. The key lessons were that complexity in implementation greatly influenced variations as policies moved from the centre to subnational levels, mostly because the response by those charged with their implementation will differ due to variations in implementation context. Another lesson is that of the role of champions, since their scope of influence and expectations vary. However, a common thread with patient needs was observed at both the national and subnational level. The timelines reflected a period when local initiatives were eclipsed by global campaigns to address stillbirth burden, especially after the launch of the 2011 Lancet stillbirth series, which to date stands out as a landmark in the global stillbirth reduction campaigns. Specifically, the authors wanted to examine variations in implementation of interventions to address stillbirth at both levels. The paper presents this anchoring discussion based on the emerging salient issues on four domains of the applied CFIR excluding one framework domain (characteristics of individuals involved). This domain can be influential in policy direction to suit particular stakeholder interests such as NGO actors as well as the undue influence of politicians during policy formulation decision-making. However, it was not explored in depth during the analysis of the current manuscript, since it had been covered extensively in two earlier publications from the same study preceding this paper [3, 24]. Outstanding features of the constructs are expounded. and they include the patient needs, intervention complexities and contextual factors, as well as the role of champions.

An important finding was the variations and influence of complexity at both levels in directing the response. The quality of available MCH services was a thorny issue for planners and implementers. At the national level, there was a tendency to overemphasize policy failures which occasioned facility-level fresh stillbirth to anchor national response. And yet at the subnational level, intertwined layers of complex issues may have accounted for stillbirth rates; for example, cases of demand-side bottlenecks beyond the referral systems were observed. Choosing to respond to known complex issues was a strategic move in anticipation of better outcomes rather than focusing entirely on recommended “magic bullets”. For example, the MoH opted to prioritize responding to risk factors for fresh stillbirth which were happening within health facilities. In addition, the fact that many of the cases were happening due to complications during delivery called for scale-up of comprehensive emergency obstetric care services while equipping available human resource with the required skills and revising staffing norms to include recruitment of rare cadres such as anaesthesiologists. This observation compares well with results from the better birth trial conducted in Utter Pradesh that established that despite the adoption of recommended practices, improved quality of facility-based childbirth and a change in provider behaviours, it did not achieve an impact on a composite outcome including stillbirth between intervention and control sites [14, 35, 36]. This reflects the amount of influence that context can exert on known high-impact interventions in determining the intervention outcomes.

Our study also established variations in national- and subnational-level champions. This could perhaps explain variations in the implementation of interventions at these levels. At the national level, these were driven by their passion for MCH and influence among peers, and were drawn from different MCH stakeholder groups including development partners, professional associations, implementers and top government top bureaucrats, among others. One common feature among all was that representation was driven by interests from each of the stakeholders. This is important because the ability to influence other stakeholders becomes a rationale for choosing whom to represent the group at the negation table. This can perhaps explain why adaptation at the national level appeared more rapidly, because different stakeholder representatives were top-level decision-makers within their groups. At the subnational level, our results revealed that the champions were in most cases defined by their official positions and roles played in addressing MCH challenges within the districts. At the final point of delivery, some were lower-level cadres like nurses and midwives with no authority to make decisions responding to chronic health systems challenges. Within health, most policy issues requiring major decision-making are directed to the attention of the centre (Ministry of Health), signifying that some role-bearers at best provide policy feedback from the subnational to national level. These findings echo earlier observations that challenges of limited decision spaces were not uncommon [20].

An important finding from our study as applied from the CFIR was the importance of considering patient needs at both the national and subnational levels, which made the resulting maternal health services more responsive. Integration of care was adopted with varying dimensions at the final point of maternal healthcare. This involved patient-centred care and respectful maternal care among others within the delivery of MCH services. Findings revealed that national-level efforts were responsive towards improving facility delivery experiences through improved quality of maternal health services by rolling out the RMNCH quality-of-care guidelines and operationalizing basic and comprehensive emergency obstetric care at different levels. This resonates well with recent findings that a focus on intervention components alone without much regard for contextual factors may not be adequate for addressing the stillbirth burden [13, 14]. Additional efforts in addressing contextual factors such as referral systems can be an important aspect in responding to stillbirth. Another aspect of maternal health to respond to women’s needs is the improvement of the quality of available emergency obstetric care services to minimize the possibility of referrals out.

In our previously published work [24], we reported on implementation-level contexts such as late referral and maternal continuum of delivery care-seeking which places the facility at the tail end of all available alternatives, which to health workers accounted for the majority of facility-level fresh stillbirths. Although women seek care early, some tend to start from other available alternatives including unskilled providers, and report very late at the facility. These scenarios present an important aspect to reflect on when designing interventions to address stillbirth, whether pursuing proven high-impact intervention effectiveness is enough and how to position context within this design thinking. Elsewhere, studies have established that context appeared to be stronger than the proposed interventions in influencing barriers and mortality outcomes [14, 35]. Planning implementation, therefore, goes beyond intervention adherence to focus on the contextual complexities surrounding such initiatives, especially where it impacts the quality of care. It also highlights the limitations of implementing potential strategies within controlled settings which may appear different from the routine standard-of-care settings where change is expected.

### Limitations

This study is not without limitations. First, the purposive sampling and the limited duration of the study may have overlooked perspectives of other key actors, and therefore the results reported here may represent a one-sided story more so from health workers directly involved in the implementation of MCH. Secondly, one construct, the characteristics of individuals involved, was not assessed. Third, the study relied on information provided by the respondents, and in the multifaceted context of MCH programme implementation, views may be subject to recall bias. Therefore, in the absence of documented experiences, we were unable to triangulate respondents’ information. Fourth, there was a risk of social desirability associated with research related to programme implementation experiences, where respondents may systematically edit out views that may appear to reflect the poor performance of the health facilities they represent. However, a key strength of this study is that the analysis and discussion draw on perspectives from multiple sources of data including both national- and subnational-level key actors in the implementation of MCH policy in the country. This enabled triangulation of information provided from more than one source, which ensured the reliability of the information collected. Besides, the analysis was conducted deductively, drawing on the main constructs of the applied framework; where particular text fitting to a particular theme of the framework was not applied, it does not reflect the weakness of the framework but the inability to access that data during the study period.

*Implications for policy* It is important to note that when designing policies for subnational-level implementation, there is no one-size-fits-all, and hence there is a need for decision space to facilitate context-specific operationalization of policies at the subnational level to fit the context.

*Implications for practice* The lesson carried from this learning is that it is always important for embedded policy evaluations to establish operational-level factors likely to lead to variations in implementation of policies from the national level to what is finally translated a the subnational level.

*Implications for future research* A recommendation for future research would be for more in-depth studies employing such methodologies as process tracing to establish points of divergence and underlying factors from national to subnational-level implementation.

## Conclusion

The CFIR facilitated the identification of variations in the implementation of similar strategies at different levels. This framework has the potential for use in identifying gaps leading to variations in implementation which may account for the inability to realize the different policy aspects and objectives across varying implementation contexts within the country. Application of the CFIR enabled the study team to break down complex implementation processes to analyse the experiences and variations from the national to subnational levels.

## Data Availability

The datasets used and/or analysed during the current study are available from the corresponding author on reasonable request.

## Abbreviations

ANC: Antenatal care
CFIR: Consolidated Framework for Implementation Research
COREQ: Consolidated Criteria for Reporting Qualitative Research
DHIS: District Health Information Software
DHMT: District health management team
ENAP: Every Newborn Action Plan
RMNCH: Reproductive, maternal, newborn and child health
MDG: Millennium Development Goals
MCH: Maternal and child health
MoH: Ministry of Health
MPDSR: Maternal and perinatal death surveillance and responses
RBF: Results-based financing
RMNCAH: Reproductive, maternal, newborn, child and adolescent health
SCC: Safe Childbirth Checklist
TBAs: Traditional birth attendants
UHC: Universal health coverage
URMCHIP: Uganda Reproductive Maternal Child Health Services Improvement Project
